# Tobacco use prevalence and its determinate factor in Ethiopia- finding of the 2016 Ethiopian GATS

**DOI:** 10.1186/s12889-022-12893-8

**Published:** 2022-03-21

**Authors:** Sisay Derso Mengesha, Kirubel Tesfaye Teklu, Abel Weldetinsae, Melaku Gizaw Serte, Moa Abate Kenea, Daniel Abera Dinssa, Mesay Getachew Woldegabriel, Tsigereda Assefa Alemayehu, Wassihun Melaku Belay

**Affiliations:** 1 Ethiopian Public Health Institute, Gulelle Patriot Street, P.O.Box 1242, Addis Ababa, Ethiopia; 2 World Health organization- Country office for Ethiopia, UNECA Compound, Zambezi Building, Addis Ababa, Ethiopia

**Keywords:** Tobacco use, Predicting factor, GATS, NCD, Ethiopia

## Abstract

**Background:**

Tobacco, one of the risk factors for non-communicable diseases, kills 8 million people each year. Like other sub-Saharan countries, Ethiopia faces the potential challenge of a tobacco epidemic. However, there is no organized data on the prevalence of tobacco use in the country. Therefore, this study aims to determine adult tobacco use in Ethiopia.

**Methods:**

The study was conducted using the WHO and CDC GATS survey methods. Complex survey analysis was used to obtain prevalence and population estimates with 95% confidence intervals. Bivariate regression analyses were employed to examine factors related to tobacco use.

**Results:**

The overall tobacco use percentage was 5.0% [95% CI (3.5, 6.9)], of which 65.8% [95% CI (53.4, 76.3)] only smoked tobacco products; 22.5% [95% CI (15.7, 31.2)] used smokeless tobacco only; and 11.8% [95% CI (6.5, 20.4)] used both smoked and smokeless tobacco products. In 2016, more men adults (8.1%) used tobacco than women did (1.8%). Eight out of eleven states have a higher smoking rate than the national average (3.7%). Gender, employment, age, religion, and marital status are closely linked to current tobacco use (*p*-value< 0.05). Men adults who are employed, married, and mostly from Muslim society are more likely to use tobacco.

**Conclusion:**

The prevalence of tobacco use is still low in Ethiopia. However, the percentage of female smokers is increasing, and regional governments such as Afar and Gambella have a relatively high prevalence. This calls for the full implementation of tobacco control laws following the WHO MPOWER packages. A tailored tobacco control intervention targeting women, younger age groups, and regions with a high proportion of tobacco use are recommended.

## Background

The growing burden of chronic non-communicable diseases worldwide including the African continent is gaining attention [1–3]. It is estimated that 41 million people die each year from non-communicable diseases, most of them in low- and middle-income countries [4]. Heart diseases, stroke, cancers, chronic respiratory diseases, and diabetes by far constitute the leading cause of mortality in the world [4, 5]. In 2030, such diseases are projected to claim the lives of 52 million people [6].

As one of the modifiable risk factors for non-communicable diseases (NCDs), tobacco is responsible for almost 8 million deaths each year of these 7 million from direct tobacco use and 1.2 million due to secondhand smoke exposure [7]. Smoking is attributed to high proportion of lung cancer (71%), chronic respiratory diseases (42%), and cardiovascular disease (10%) [8]. Globally, tobacco use causes more than one and half trillion dollars of economic damage each year [7]. If the current trend is not curbed, it will be responsible for 1 billion deaths at the end of this century [9]. This NCDs epidemics is fueled by a combination of risk factors, including tobacco use, unhealthy diet, lack of physical activity, harmful alcohol use, overweight or obesity, and elevated blood pressure, blood sugar, and cholesterol [10].

In contrary to the common perception that NCDs are only a problem of the richer world, but 80% of chronic disease deaths occur in low and middle-income countries, and affect younger populations and lead to premature mortality [11]. This inequality of burden of NCD may be due to lack of prevention or effective management of the diseases in developing country [12, 13]. Thus the low- and middle-income countries are affected by the double burden of the growing chronic diseases and communicable diseases, maternal and perinatal conditions, and nutritional problems [14].

Ethiopia like other African countries experiences the challenges of a potential tobacco epidemic. According to the Global Burden of Disease 2016 report, annually about 16,800 people (259 males and 65 females per week) in Ethiopia die from tobacco-related death which is relatively higher compared to other African countries such as Kenya, Cameroon, and Botswana [15].

Evidence around tobacco smoking in Ethiopia is meager. The Ethiopian Demographic and Health Survey (EDHS) and NCD STEPS survey generated the only nationally representative evidence on tobacco use with limited indicators [16, 17]. Besides, Global Youth Tobacco Survey (GYTS,2005) conducted in Secondary Schools in Addis Ababa is the second organized data on youth tobacco use in Ethiopia [18]. However, comprehensive, evidence-based population-level data on tobacco use was not available to show the magnitude, trends, and impact of the tobacco epidemic in the country. Hence, the Ethiopian Global Adult Tobacco Survey (GATS) 2016 measuring tobacco use, frequency of smoking, type of tobacco products and other tobacco control indicators at national level was conducted to address this major gap at national level. In addition, this paper was examined the association between key individual sociodemographic characteristics and current tobacco use. The present study also complements other government agency like Central Statistical Agency (CSA) efforts in periodically monitoring the tobacco epidemic and provides comprehensive evidence and information for tobacco control planning and policy development.

## Methodology

### Study setting and tools

Ethiopian GATS 2016 was conducted as nationally representative household survey targeting adults both men and women aged 15 years or older residing in any of the nine regional states and two city administrations in Ethiopia. The sample selection did not include institutionalized adults. The survey was conducted in the target population with a usual member of the sampled household who either (1) did not have any other residence, or (2) had multiple residences but had been living in the sampled household for at least six months during the year prior to the survey. The institutional population living in prisons, hospitals, military barracks, school dormitories, etc. were excluded from the universe defined for the household surveys [19].

The GATS core questionnaire was adapted for Ethiopia to include some optional questions through a process of intensive consultations to reflect country-specific questions, meetings and proposed edits by the GATS Questionnaire Review Committee (QRC). Recognizing the high level of population diversity in Ethiopia—multiple nationalities and ethnicities, varying cultures, and 80 languages spoken—the Ethiopian GATS committee came to consensus to make use of three primary languages for interviewing: Amharic, Oromiffa, and Tigrigna. In addition, the English questionnaire was also included in the survey. To address language barriers, the committee recruited interviewers and supervisors who have skills in speaking and writing these languages. The QRC approved and incorporated the pretest experience into the questionnaire.

### Survey design and sampling procedures

Researchers conducted a population-based descriptive cross-sectional study using the World Health Organization (WHO) and US Centers for Disease Control and Prevention (CDC) GATS globally standardized protocol to determine adult tobacco use and other tobacco control indicators in Ethiopia [20]. The sampling frame was based on the population and housing census conducted in 2007 [21]. A multi-stage geographically stratified cluster sampling and Population Proportion to size (PPS) designs were used to produce estimate for key tobacco control indicators for the country as a whole and by gender and residence (urban or rural). In addition, the sub-national prevalence was generated at the level of regions (9 regional states and two autonomous cities) without further disaggregation by age, gender, and place of residence. The GATS sampling procedure followed three stage approach and in the first stage 375 enumeration areas (EAs) — i.e., primary-sampling units (PSUs) — were selected from the master sample using probability proportion to size (PPS). An equal number of PSUs i.e. EAs were allocated to urban and rural domains before selection. Prior to selecting the household sample, a re-enumeration process (mapping and listing) of all 375 GATS EAs was conducted to update the household address information. The process of re-enumeration allowed for complete household coverage with precise sampling results for the survey. In the second stage, 10,875 households were chosen systematically from selected PSUs/EAs (secondary sampling unit). Twenty-nine households were selected per PSU/EA. In the last stage, one eligible member 15 years of age or older was selected randomly from the list (roster of 15+ eligible individuals) using handheld devices within each selected household.

A total of 10,875 households were sampled and of these 10,649 households completed the survey and 10,150 individuals were successfully interviewed (one individual was randomly chosen from each selected household to participate in the survey). The total response rate for Ethiopian GATS was 93.4%. The household response rate was 97.9% (98.1% urban, 97.9% rural), while the individual response rate was 95.4% (95.8% urban, 95.0% rural).

### Study variables

The dependent variable “current tobacco use” is constructed based on the responses provided to the GATS individual questions in both sexes-male and female. The study population were asked if they were currently use both smoking and smokeless tobacco products. Smoking tobacco products including shisha, cigar, gaya (local traditional smoking tobacco leaves) and others. Current tobacco use in this manuscript includes daily and occasional (less than daily) smokers and smokeless tobacco users. We used demographic variables such as gender, age, educational level, marital status, occupation, wealth index (5 levels), and religion as smoking predicting factors. In this article, the wealth index is a measure of a family’s overall standard of living, measured by the size of assets, such as vehicles, television, radio, basic water and sanitation facilities, and land. Moreover, we used wealth index as equivalent of socio-economic status through this paper.

### Data analysis

Complex survey data analysis adapted from GATS data analysis manual [22, 23] was used to obtain prevalence and population estimates with 95% confidence intervals. To improve the representativeness of the sample in terms of the size, distribution, and characteristics of the study population, sample weights were calculated for each respondent before the analysis. SPSS version 19, SAS version 9.2, and SUDAAN version 10.1 software were used for data analysis. Standard errors were calculated using Taylor series linearization.

Multivariate analysis of risk factor for cigarette smoking was conducted to select predictors of any tobacco use in Ethiopia. Measures of associations (odds ratio) are also applied to determine the prevalence of cigarette smoking and factors associated with it. Additionally, bivariate and multivariate analyses were conducted to determine odds ratios and confidence intervals.

Statistical significance was measured by comparing the 95% confidence intervals of two estimates to determine whether they were statistically different. This report states two estimates are different, either higher or lower, only if their confidence intervals are non-overlapping.

## Result

### Sample and population characteristics

In the 2016 GATS survey, the total unweighted sample was 10,150. Based on the 2007 Ethiopia population census, the weighted number of adults aged 15 years or above was 68.37 million [24].

The distribution of the unweighted sample by gender shows that 4627 men and 5523 women completed the survey, with the weighted proportions by gender showing 49.9% (34.1 million) for men and 50.1% (34.25 million) for women. By residence, the number of unweighted respondents was 5064 for urban areas and 5086 for rural areas. The weighted population in urban areas was 24.1% (16.5 million) and 75.9% (51.9 million) for rural areas [24]. Distribution by age group indicates that the number of unweighted respondents was 2754 for ages 15–24 years; 5341 for 25–44 years; 1576 for 45–64 years; and 483 for age 65 years and over. The weighted percentages for these age groups were 45.3, 36.1, 14.3, and 4.2% for age groups 15–24, 25–44, 45–64, and 65 years or more respectively. The weighted percentage with no formal education was 35.7%; primary school completed was 37.0%; secondary school completed was 21.3%, and higher than secondary education was 6.0% (Table 1).

**Table 1:** Distribution of adults ≥15 years old by selected demographic characteristics – Ethiopian GATS, 2016

Demographic Characteristics	Weighted			Unweighted Number of Adults
	Percentage(95% CI)		Number of Adults (in thousands)	
Overall	100		68,371.8	10,150
*Gender*				
Male	49.9	(48.1, 51.8)	34,147.2	4627
Female	50.1	(48.2, 51.9)	34,224.5	5523
*Age (years)*				
15–24	45.3	(43.0, 47.7)	30,995.9	2750
25–44	36.1	(34.2, 38.1)	24,692.7	5341
45–64	14.3	(12.9, 15.8)	9795.0	1576
65+	4.2	(3.5, 5.1)	2888.2	483
*Residence*				
Urban	24.1	(22.1, 26.3)	16,503.4	5064
Rural	75.9	(73.7, 77.9)	51,868.4	5086
*Education Level*				
No formal education	35.7	(32.8, 38.7)	24,399.9	3768
Primary	37.0	(34.9, 39.1)	25,300.9	3195
Secondary	21.3	(19.4, 23.4)	14,577.8	2098
Higher than secondary	6.0	(5.0, 7.1)	4084.0	1070
*Wealth Index*				
Lowest	38.0	(33.7, 42.5)	25,975.4	2324
Low	26.2	(23.0, 29.7)	17,943.4	2016
Middle	13.9	(11.5, 16.7)	9498.0	1900
High	11.2	(9.4, 13.3)	7652.3	1924
Higher	10.7	(9.1, 12.4)	7302.7	1986

### Prevalence of tobacco use, smoking frequency, and type of smoking products

Table 2 shows the percentage distributions of Ethiopian adults by current smoking status. An estimated 3.7% (95% CI = 2.5–5.0) of Ethiopian adults smoked tobacco in some form in 2016. Of that number, more than eight in ten (86% of all adults) smoked daily and less than two in ten (14% of all adults) were occasional smokers. In terms of gender, about 6.2% of men and 1.2% women adults were current smokers. The study also presented the distribution of adult smokers by smoking frequency including daily, occasional, and non-smoker. According to this, 3.2% of Ethiopian adults smoke daily, 0.5% occasionally smoke, and the remaining 96.3% were non-smokers. Among men 5.2%, were daily smokers, 0.9%, were occasionally smokers, and 93.8% were non-smokers. The proportion of women who were daily women smokers, occasional smokers, and non-smokers was 1.1, 0.1 and 98.8%, respectively.

**Table 2:** Percentage of adults ≥15 years old, by detailed smoking status and gender – Ethiopian GATS, 2016

Smoking Status	Overall		Male		Female	
	*Percentage (95% CI)*					
**Current tobacco smoker**	3.7	(2.7, 5.0)	6.2	(4.8, 7.9)	1.2	(0.5, 3.1)
Daily smoker	3.2	(2.3, 4.5)	5.2	(4.0, 6.9)	1.1	(0.4, 3.0)
Occasional smoker	0.5	(0.3, 0.8)	0.9	(0.6, 1.5)	0.1	(0.0, 0.3)
Occasional smoker, formerly daily	0.1	(0.1, 0.3)	0.3	(0.2, 0.5)	0.0	(0.0, 0.1)
Occasional smoker, never daily	0.4	(0.2, 0.6)	0.7	(0.4, 1.1)	0.1	(0.0, 0.3)
**Non-smoker**	96.3	(95.0, 97.3)	93.8	(92.1, 95.2)	98.8	(96.9, 99.5)
Former daily smoker	1.2	(0.8, 1.8)	1.9	(1.3, 2.6)	0.5	(0.2, 1.1)
Never daily smoker	95.1	(93.7, 96.2)	91.9	(90.0, 93.5)	98.3	(96.6, 99.2)
Former occasional smoker	0.5	(0.3, 0.8)	0.8	(0.5, 1.3)	0.1	(0.1, 0.3)
Never smoker	94.7	(93.2, 95.8)	91.1	(89.2, 92.7)	98.2	(96.5, 99.0)
**Current smokeless tobacco user**	1.7	(0.9, 3.0)	2.6	(1.5, 4.3)	0.8	(0.2, 2.3)
Daily user	1.5	(0.8, 2.7)	2.3	(1.3, 4.1)	0.6	(0.2, 1.9)
Occasional user	0.2	(0.1, 0.4)	0.3	(0.1, 0.6)	0.1	(0.0, 0.5)
Occasional user, formerly daily	0.1	(0.0, 0.2)	0.1	(0.0, 0.3)	0.0	(0.0, 0.1)
Occasional user, never daily	0.1	(0.1, 0.3)	0.2	(0.1, 0.4)	0.1	(0.0, 0.5)
**Non-user of smokeless tobacco**	98.3	(97.0, 99.1)	97.4	(95.7, 98.5)	99.2	(97.7, 99.8)
Former daily user	0.2	(0.1, 0.4)	0.3	(0.1, 0.6)	0.1	(0.0, 0.4)
Never daily user	98.1	(96.8, 98.9)	97.1	(95.4, 98.2)	99.2	(97.7, 99.7)
Former occasional user	0.1	(0.0, 0.2)	0.1	(0.1, 0.3)	0.0	(0.0, 0.1)
Never user	98.1	(96.7, 98.9)	97.0	(95.2, 98.1)	99.1	(97.7, 99.7)

Current tobacco smokers includes both daily and occasional (less than daily) smokers of any tobacco products including manufactured and hand roll, Gaya, etc

Daily smokers includes smokers who smoke any type of smoking tobacco products in daily bases

Occasional smokers refers to less than daily smokers of any type of smoking tobacco products

**Fig. 1:**
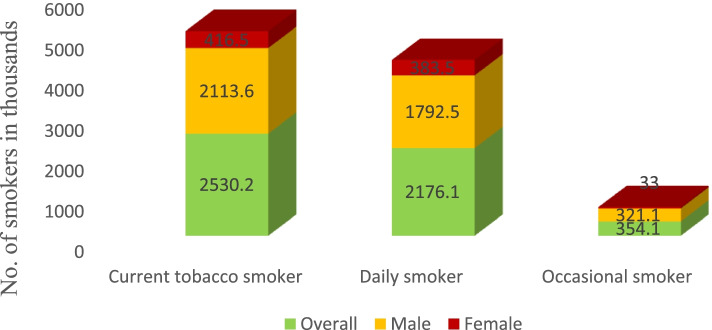
Number of adults ≥15 years old, by detailed smoking status and gender – Ethiopian GATS, 2016

Using this prevalence, we estimated a weighted number of users of any tobacco product classified by detailed smoking status and gender (Fig. 1). There were 2,530,200 tobacco smokers aged 15 years or above in Ethiopia. Of this group, 2,113,600 were men (3.7%) and 416,500 were women (1.2%). Ethiopian GATS estimated the number of daily tobacco smokers to be 2,176,100 (1,792,500 men and 383,500 women). In addition to these daily tobacco smokers, an estimated 354,100 adults smoked tobacco occasionally (0.5%).

In addition to smoking tobacco products, many Ethiopians use smokeless tobacco products. Overall, 1.7% (95% CI = 0.9–3.0) of adults aged 15 years or older used smokeless tobacco. By gender, 2.6% (95% CI = 1.5–4.3) of men and 0.8% (95% CI = 0.2–2.3) of women used smokeless tobacco. The majority of smokeless tobacco users were daily users (1.5% (95% CI = 0.8–2.7) compared to occasional users (0.2% (95% CI = 0.1,0.4).

Among non-smokers, 1.2% (95% CI = 0.8–1.8) of Ethiopian adults were former daily smokers (1.9% of males and 0.5% of females), and 0.5% were former occasional smokers. The majority of Ethiopian adults (94.7%) had never smoked tobacco in their lifetime; 91.1% of men and 98.2% of women (Table 2).

While among non-user of smokeless tobacco, 0.2% (95% CI = 0.1–0.4) of Ethiopian adults were former daily users (0.3% of males and 0.1% of females), and 0.1% were former occasional users. The majority of Ethiopian adults (98.1%) had never used smokeless tobacco in their lifetime; 97.0% of men and 91.1% of women (Table 2).

The overall prevalence of current tobacco users (including daily and occasional tobacco smokers and all smokeless tobacco users) was 5%, of whom 65.8% only smoked tobacco products; 22.5% used smokeless tobacco only, and 11.8% used both smoked and smokeless tobacco products.

More than six in ten men (67.6%) who were current tobacco users smoked tobacco only; 21.3% used smokeless tobacco only, and 11.1% used both smoked and smokeless tobacco. However, more than half of women (57.3%) who were current tobacco users used smokeless tobacco, while 27.8% used smoked tobacco. The percentage of women tobacco users who both smoked and used smokeless tobacco was 14.8%. The use of smokeless tobacco was higher among rural tobacco smokers (25.8%) than among urban tobacco users (6.95%). Besides, rural smokers used both smoked and smokeless tobacco products more than urban smokers did (13.3% vs. 4.2%) (Table 3).

**Table 3:** Percentage distribution of current tobacco users ≥15 years old, by tobacco use pattern and selected demographic characteristics – Ethiopian GATS, 2016

Demographic Characteristics	Current Tobacco Users^a^		Type of Current Tobacco Use						
			Smoked only		Smokeless only		Both smoked and smokeless		Total
	*Percentage (95% CI)*								
**Overall**	5.0	(3.5, 6.9)	65.8	(53.4, 76.3)	22.5	(15.7, 31.2)	11.8	(6.5, 20.4)	100
*Gender*									
Male	8.1	(6.2, 10.6)	67.6	(54.9, 78.2)	21.3	(13.4, 32.2)	11.1	(6.3, 18.7)	100
Female	1.8	(0.7, 4.3)	57.3	(39.5, 73.5)	27.8	(17.2, 41.7)	14.8	(6.2, 31.5)	100
*Residence*									
Urban	3.8	(2.9, 5.0)	88.9	(81.3, 93.6)	6.9	(3.7, 12.4)	4.2	(1.9, 9.3)	100
Rural	5.3	(3.5, 8.0)	60.9	(47.6, 72.7)	25.8	(18.1, 35.3)	13.3	(7.2, 23.4)	100
*Wealth Index*									
Lowest	5.9	(3.2, 10.5)	47.6	(34.5, 61.1)	32.5	(23.5, 42.9)	19.9	(11.2, 32.9)	100
Low	5.2	(3.4, 8.0)	74.4	(58.3, 85.8)	19.6	(10.0, 34.9)	5.9	(2.2, 15.1)	100
Middle	3.7	(2.3, 6.1)	83.9	(65.7, 93.4)	12.9	(5.6, 26.9)	3.2	(0.7, 13.3)	100
High	3.6	(2.4, 5.4)	88.3	(77.2, 94.3)	6.6	(2.9, 14.5)	5.1	(1.7, 14.3)	100
Higher	4.0	(2.8, 5.7)	94.6	(87.5, 97.8)	3.0	(1.5, 6.1)	2.4	(0.4, 12.9)	100

^a^Includes daily and occasional (less than daily) smokers or smokeless users

In Ethiopia, 3.7% (95% CI = 2.7, 5.0) of adults were current smokers of any smoked type of tobacco products and of these 2.9% (95% CI = 2.2, 3.7) smoked any cigarettes (includes manufactured and hand rolled cigarettes), and 0.7% (95% CI = 0.4, 1.0) smoked other types of tobacco products including pipes full of tobacco, cigars and any other reported smoking tobacco products. Among adult cigarette smokers aged 15+ years, 2.7% (95% CI = 2.1, 3.5) smoked manufactured cigarettes and 1% (95% CI = 0.7, 1.4) smoked hand-rolled cigarettes (Table 4).

**Table 4:** Percentage of adults ≥15 years old who are current smokers of various smoked tobacco products, by wealth index – Ethiopian GATS, 2016

Demographic Characteristics	Any smoked tobacco product		Any cigarette^a^		Type of Cigarette				Gaya		Water pipe		Other smoked tobacco^b^	
					Manufactured		Hand-rolled							
	*Percentage (95% CI)*													
**Overall**	3.7	(2.7, 5.0)	2.9	(2.2, 3.7)	2.7	(2.1, 3.5)	1.0	(0.7, 1.4)	1.0	(0.4, 2.4)	0.3	(0.1, 0.6)	0.7	(0.4, 1.0)
**Residence**														
**Urban**	3.4	(2.5, 4.5)	3.2	(2.4, 4.3)	3.2	(2.3, 4.3)	1.1	(0.8, 1.6)	0.4	(0.2, 0.7)	0.2	(0.1, 0.6)	0.6	(0.3, 1.0)
*Rural*	3.8	(2.6, 5.5)	2.8	(2.0, 3.8)	2.6	(1.8, 3.6)	1.0	(0.6, 1.6)	1.2	(0.4, 3.1)	0.3	(0.1, 0.8)	0.7	(0.4, 1.1)
*Wealth Index*														
Lowest	3.8	(2.1, 6.8)	2.0	(1.3, 3.0)	1.8	(1.2, 2.7)	0.8	(0.4, 1.6)	2.0	(0.7, 5.6)	0.1	(0.0, 0.4)	0.5	(0.3, 0.9)
Low	4.1	(2.6, 6.2)	3.8	(2.4, 5.9)	3.5	(2.1, 5.8)	0.7	(0.4, 1.3)	0.5	(0.2, 1.1)	0.1	(0.0, 0.5)	0.8	(0.4, 1.9)
Middle	3.1	(1.8, 5.3)	3.0	(1.7, 5.3)	3.0	(1.7, 5.3)	1.4	(0.5, 3.9)	0.3	(0.1, 0.7)	0.9	(0.2, 4.3)	1.1	(0.3, 4.0)
High	3.1	(2.0, 4.8)	3.1	(2.0, 4.8)	3.1	(2.0, 4.8)	1.4	(0.7, 2.9)	0.1	(0.0, 0.1)	0.2	(0.1, 0.4)	0.3	(0.1, 1.0)
Higher	3.7	(2.6, 5.3)	3.4	(2.3, 5.0)	3.4	(2.3, 4.9)	1.5	(0.9, 2.4)	0.4	(0.1, 1.2)	0.8	(0.4, 1.6)	0.6	(0.2, 1.4)

Current smokers includes both daily and occasional (less than daily) smoker

^a^ Includes manufactured and hand rolled cigarettes

^b^ Includes pipes full of tobacco, cigars and any other reported smoking tobacco products

Wealth index is a measure of a family’s overall standard of living, measured by the size of assets, such as vehicles, television, radio, basic water and sanitation facilities, and land

The present study also shows the distribution of a variety of smoking tobacco products by wealth index. All economic groups consumed all types of tobacco products, including manufactured cigarettes. About 0.9% (95% CI = (0.2, 4.3) of the middle wealth index group and 0.8% (95% CI = (0.4, 1.6) of the higher wealth index groups were used water pipe products, respectively. On the other hands, about 2.0% (95% CI = (0.7, 5.6) the lowest and 0.5% (95% CI = (0.2, 1.1) the low wealth index groups were used other smoked tobacco.

By place of residence, there was a difference between urban and rural residents who used any tobacco product (3.4% (95% CI = 2.5, 4.5), and 3.8% (95% CI = 2.6, 5.5), respectively, mainly attributing to a slightly higher level of use of Gaya, water pipe, and other forms of tobacco (Table 4). There were no significant differences among users of cigarettes between urban and rural (3.2% (95% CI = 2.4, 4.3) and 2.8% (95% CI = 2.0, 3.8), respectively.

**Fig. 2:**
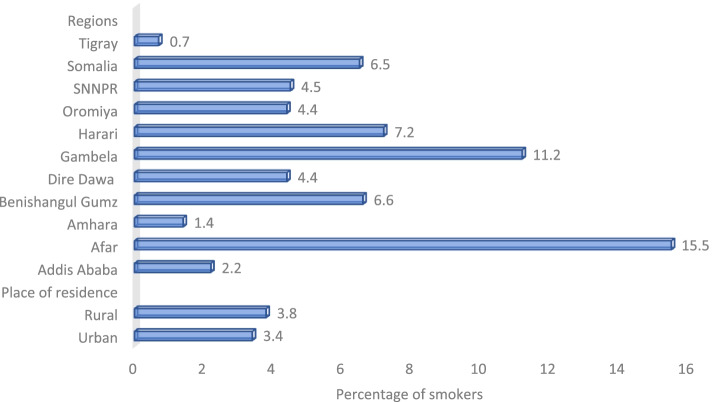
Percentage of adults ≥15 years old who are current smokers of any smoked tobacco products, by place of residence – Ethiopian GATS, 201

Across regions, percentages of people who smoked any tobacco product ranged from 0.7% in Tigray to 15.5% in Afar. Eight regions, including Afar 15.5% (95% CI = 10.2, 22.9), Gambella 11.2% (95% CI = 7.2, 17.0), Harrari 7.2% (95% CI = 5.4, 9.6), Benushangul Gumz 6.6% (95% CI = 3.6, 11.8), Somalia 6.5% (95% CI = 4.8, 8.8), Oromiya 4.4% (95% CI = 3.0, 6.4), SNNPR 4.5% (95% CI = 1.8, 10.9, and Dire Dawa 4.4% (95% CI = 3.0, 6.2) had higher smoking rates than the national estimate of 3.7% (95% CI = 2.7, 5.0) (Fig. 2).

### Average age at initiation of daily smoking and distribution of initial age

**Fig. 3:**
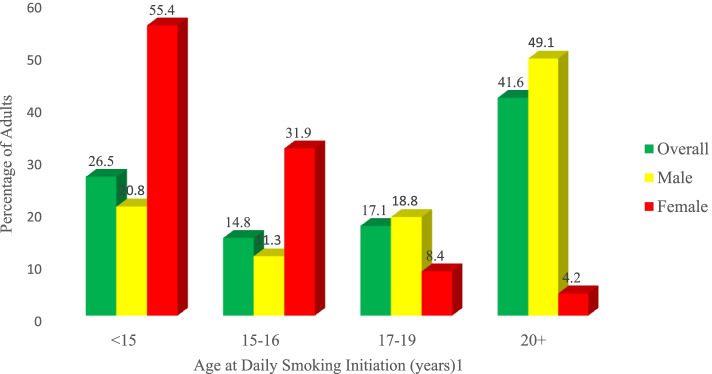
Percentage distribution of ever daily smokers 20–34 years old by age at daily smoking initiation by gender, Ethiopian GATS 2016

Among daily cigarette smokers aged 20–34 at the time of the survey, 26.5% started smoking daily before the age of 15; 14.8% started at age 15–16; 17.1% started at age 17–19; and 41.6% started at age 20 or older (Fig. 3).

#### Number of manufactured cigarettes smoked per day

The number of cigarettes smoked per day (including manufactured and hand-rolled cigarettes) is a key indicator in determining nicotine dependence [25]. Daily cigarette smokers in Ethiopia smoked on average 10.4 manufactured cigarettes per day. Overall, 28.8% of daily cigarette smokers smoked fewer than five cigarettes a day; 13.4% smoked five to nine per day; 12.9% smoked 10–14 per day; 22.5% smoked 15–24 per day, and 22.4% smoked 25 or more cigarettes per day (Table 5).

**Table 5:** Average number and percentage distribution of cigarettes smoked per day among daily cigarette smokers ≥15 years old, by gender and selected demographic characteristics – Ethiopian GATS, 2016

Demographic	Distribution of the number of cigarettes smoked on average per day^a^									
Characteristics	< 5		5–9		10–14		15–24		≥25	
	*Percentage (95% CI)*									
**Overall**	28.8	(18.4, 42.1)	13.4	(7.4, 23.0)	12.9	(7.8, 20.5)	22.5	(14.3, 33.8)	22.4	(15.1, 31.9)
*Gender*										
Male	28.8	(18.1, 42.5)	13.6	(7.4, 23.6)	12.2	(7.3, 19.8)	22.6	(14.1, 34.3)	22.8	(15.3, 32.5)
Female	29.9	(8.3, 66.8)	8	(2.3, 24.4)	28.8	(5.4, 74.3)	20.2	(5.7, 51.3)	13.1	(4.8, 30.9)
*Residence*										
Urban	25.9	(14.4, 41.9)	13	(6.0, 25.9)	11.5	(5.3, 23.1)	17.2	(11.3, 25.2)	32.4	(23.7, 42.6)
Rural	29.8	(17.0, 46.8)	13.5	(6.3, 26.4)	13.3	(7.2, 23.4)	24.4	(13.9, 39.1)	19	(10.9, 31.1)
*Wealth Index*										
Lowest	17.4	(6.6, 38.8)	14.4	(5.9, 31.0)	10.5	(4.6, 22.5)	31.6	(16.0, 52.8)	26.1	(13.9, 43.6)
Low	47.9	(26.8, 69.9)	15.7	(6.1, 34.7)	15.6	(6.0, 35.1)	11.9	(4.9, 26.0)	8.8	(3.7, 19.6)
Middle	20.7	(7.1, 47.0)	2.5	(0.8, 7.6)	14.4	(5.1, 34.4)	43.7	(17.2, 74.4)	18.7	(7.4, 40.0)
High	20.1	(9.0, 39.1)	18.6	(7.0, 41.1)	10.4	(2.5, 34.3)	16	(6.5, 34.2)	34.8	(20.2, 53.0)
Higher	13.3	(5.0, 31.2)	13.4	(5.1, 30.8)	9.6	(2.5, 30.7)	12.1	(4.8, 27.5)	51.5	(37.0, 65.8)

^a^Among daily cigarette smokers. Cigarettes include manufactured and hand-rolled

Number of manufacture cigarette smoker per day (mean ± sd) 10.4 ± 12.78

By residence, about 32.4% (95% CI: 23.7, 42.6) those in urban areas smoked on average more than 25 cigarettes per day compared with 19.0% (95% CI: 10.9, 31.1) those in rural areas smoked the same number of cigarettes per day. In addition, the number of cigarettes smoked per day in Ethiopia varied by type of wealth index, and approximately 48.0% (95% CI: 26.8, 69.9) of low-wealth index group smoked less than five cigarettes per day, and a high proportion of higher-wealth index group about 51.5% (95% CI: 37.0, 65.8) smoked more than 25 cigarettes per day (Table 5).

### Predictors of any tobacco use in Ethiopia

To identify the independent factors associated with tobacco use, variables including gender, age, place of residence, education level, occupational, wealth index, religion and marital status were analyzed. We used the lowest percentage as a reference for each group in each factor. The bivariate analysis indicated that gender, age, marital status, and occupation showed a significant association with current tobacco use at *p*-value> 0.05. However, place of residence, wealth index, and education were not significantly associate with current tobacco use (Table 6).

**Table 6:** Predictors of any tobacco use in Ethiopia-Ethiopian GATS, 2016

Demographic Characteristics	COR	95% CI	***P***-value
***Gender***			0.0017
Male	7.63	(2.16,26.96)	
Female	1		
***Age (years)***			0.0005
15–24	1		
25–44	2.86	(1.51, 5.41)	
45–64	4.54	(2.25,9.15)	
65+	3.55	(1.41, 8.91)	
***Residence***			0.4471
Urban	1		
Rural	1.32	(0.64, 2.74)	
***Education Level***			0.2875
No formal education	1.5	(0.76, 2.97)	
Primary	1.88	(0.99,3.58)	
Secondary	1.24	(0.61, 2.49)	
Higher than secondary	1		
***Occupation***			< 0.05
Employed	8.86	(3.11,25.20)	
Retired or unemployed	5.46	(1.69, 17.64)	
Homemaker	1.54	(0.45, 5.30)	
Student	1		
***Wealth Index***			0.8791
Lowest	1.03	(0.46,2.32)	
Low	1.29	(0.64,2.59)	
Middle	0.88	(0.44,1.79)	
High	0.91	(0.53,1.57)	
Higher	1		
***Religion***			< 0.05
Muslim	2.89	(0.95,8.82)	
Christian	1.49	(0.57,3.88)	
Other	1		
None	101.8	(36.11,287.04)	
***Marital Status***			0.0102
Single	1.68	(0.61,4.64)	
Married	3.98	(1.39,11.43)	
Cohabiting	–	–	
Separated	3.47	(1.09,10.98)	
Divorced	1.51	(0.48,4.75)	
Widowed	1		

*COR* crude odd ratio

## Discussion

Ethiopian GATS was the very first of its kind in Ethiopia, and it provided critical information on key tobacco control indicators for policymakers and the tobacco control community. Before GATS, only DHS and NCD STEPS surveys reported the prevalence of tobacco use in Ethiopia at a national level. However, these surveys did not address tobacco use in full detail as GATS did. The present study provide estimates with confidence intervals on tobacco use prevalence and type of products smoked as well as predictors of any tobacco use (Table 6) among adults in Ethiopia.

Even though tobacco is one of the condemned products by most Ethiopian cultures [26], it has been used as traditional medicine in some parts of the country [27]. As growing evidence indicates, the practice of tobacco use by Ethiopian adult is increasing from time to time. For instance, Ayana and his colleagues found that the prevalence of current tobacco smoking was significantly higher in the years between 2014 and 2017 than in the year before 2014 [28].

As Ethiopian GATS indicated, the overall current tobacco use prevalence of adults age 15 years and above was about 5.0% (men 8.1%, women 1.8%) in 2016 (Tables 3 and 7). This prevalence is relatively higher than other findings of DHS 2016 that reported 4.0% of men and 1.0% women smoked any type of tobacco products and STEPS 2015 that indicted 7.3% men and 0.4% women were used any form of tobacco [17, 29]. On the other hand, the smoking prevalence in Ethiopia (3.7%) is lower than most African countries such as Kenya (13.5%), South Africa (9.6%), Uganda (9.2%), Nigeria (5.5%), and others [30–33]. The tobacco industry may contribute to the observed difference in smoking prevalence, as the government-owned the National Tobacco Enterprise in Ethiopia at the time of study and the European industry operated manufacturing and cultivation of tobacco in the above-mentioned African countries such as Kenya, and Uganda. However, the absolute number indicated that 3.4 million Ethiopian adults currently use any form of tobacco and most of them smoked tobacco daily (Table 7). This indicated that tobacco use is a public health concern in the country.

**Table 7:** Distribution of current tobacco users ≥15 years old, by tobacco use pattern and gender – Ethiopian GATS, 2016

Type of tobacco use status		Overall	Male	Female
Current	Tobacco use	3.4 million	8.1%	1.8%
	Tobacco smokers	2.5 Million	6.2%	1.2%
	Cigarette smokers	1.98 Million	5.5%	0.2%
	Smokeless tobacco users	1.2 million	2.6%	0.8%
Daily	Tobacco smokers	2.2 million	5.2%	1.1%
Occasionally	Tobacco smokers	0.34 million	0.9%	0.1%

Even though low smoking prevalence of female (1.2%) comparing with male (6.2%), the proportion of women smokers are in alarming trend in Ethiopia by comparing it with previously conducted studies of DHS that only deal on women of reproductive age groups. As DHS indicates, in 2011, there were only 35 women in number who were smoked tobacco products, but in 2016 about 0.8% of women were smoked any tobacco products higher than 2011 survey. However, the present study that includes all women 15+ years found that 1.2% female adults smoked any form of tobacco products in 2016. The increment of smokers particularly among woman will affect the low quality of life and challenge the health service, as most women are responsible for childcare in Ethiopia. Tobacco use itself is a source of health inequality and it may affect women’s survival advantage over men [34].

Smoking initiation is one of the determinant of factor for long-term smoking [35], tobacco dependency and overall risk of tobacco use [36]. Early smoking initiation increases risks of experiencing smoking-related morbidities and all-cause mortality [37]. The present study revealed that approximately 3 out of 5 cigarette smokers aged 20–34 had started smoking daily before the age of 20. Among the age group of 20–34, more females (55.4% (95% CI = 26.6, 81.0) than their counter (20.8% (95% CI = 10.5, 36.8) were started smoking daily before the age of 15. Most adults start smoking before the age of 20, for example above 70% of adults in Europe started smoking daily before the age of 18 [38]. This calls to implement smoking initiation reduction among youths to protect this nicotine vulnerable groups and the overall adverse health effect of smoking [39]. Among effective interventions of tobacco control, tailored education or youth focused tobacco education and counseling by health care providers are recommended [40–42]. In our context, the term youth refers to members of society between the ages of 15–29, as defined by Ethiopian youth policy [43].

The majority of adult tobacco users were used smoked tobacco products (3.7% or 2.5 million) than smokeless tobacco products (1.7% or 1.2 million). Overall, 1.98 million or 2.9% (95% CI = 2.2, 3.7) adults (5.5% of male and 0.2% of female) smoked cigarettes in 2016 (Table 7). Among smoked tobacco products, Gaya (traditionally smoked by burring tobacco leaf over a fire and sucking it using a bamboo stick) was common in Gambella, Southern Nations, Nationalities, and People’s Region (SNNPR), and Benshangul Gumz. This may be related to cultural believes that smoking tobacco can prevent communicable diseases like malaria [17, 27]. Though Ethiopia prohibited the smoking of shisha products [44, 45], water pipes were smoked more in the Eastern part of the country than in other regions. Besides geographical variations, the pattern of smoked tobacco products depended on the economic status of the smoker. High economic groups smoked relatively more manufactured cigarettes than the lowest economic groups. Contrarily high percentage of lowest economic group smoked Gaya (Table 4).

The majority of daily smokers smoked on average 10.4 cigarettes per day in 2016. The number of cigarettes smoked per day (including manufactured and hand-rolled cigarettes) is a key indicator in determining nicotine dependence [46, 47] as well as consumption of a high number of cigarette per day could lead to different health concern such as Intracranial Aneurysm Rupture [48] and low birthweight [49]. As Hackshaw et al. (2018) and Pan et al. (2019) suggest even low cigarette consumption could lead to the risk of coronary heart diseases and stroke [50, 51].

In addition to the descriptive analysis, we examined the factors related to tobacco use in adults by using Ethiopian GATS data to understand which socio-demographic variables (education, age group, wealth index, etc.) affect the tobacco use in the country (Table 6). Based on the bivariate analysis, gender, occupation, age, and marital status are significantly associated with the current tobacco use (*p*-value< 0.05). Gender is one of the predicting factors of tobacco use as male is 7.63 times more likely to use any form of tobacco than female [COR = 7.63 95% CI (2.16–26.96). Our finding that males are seven times more likely to use any type of tobacco is consistent with other studies conducted in Ethiopia such as Guliani and colleagues’ observation [52] and similarly, findings from Defar and his colleagues indicated that males are ten times more likely to smoke tobacco than female [17]. This is also in agreement with other findings from Yemen [53] and other East Africa countries and Madagascar [54]. This finding supports the sex difference in tobacco use could be explained by some variables that contribute to psychological challenges like low social values and norms of the Ethiopia community to females [52] and by biological factors such as nicotine sensitivity, nicotine metabolism, and distribution [55].

Religion is one of the predicting factors for tobacco use and the present study found statistically significant among different religious groups (p- < 0.05). Nonbelievers (OR 101.8 CI (36.11, 287.04) and Islamic religion followers (OR 2.89 CI (0.95, 8.82) were more likely to use tobacco as compared to Christian religion followers (OR1.49 CI (0.57, 3.88). This may be related to the place of residence as most Muslim communities live in East part of the country [56] where smoking prevalence was higher and they may have exposure to smuggling tobacco products as this part of the country have high rate of illicit tobacco market share [57]. This result is consistent with other studies conducted in Ethiopia [52, 58].

In addition, age group 45–64 years OR 4.54, 95% CI (2.25, 9.15); 65+ years OR 3.55, 95% CI (1.41, 8.91); 25–44 years OR 2.86, 95% CI (1.51, 5.41), were more likely to consume tobacco than the younger age group (15–24 years). The younger age groups [15–24] are less likely to use tobacco products than all age groups above 25 years. This is consistent with other studies conducted across African countries as older age groups are more likely to smoke cigarette than younger age groups [58, 59]. This could be explained by age of initiation of smoking for continued tobacco use as a result not try to quit because they have been smoking for a long time and think that it will not cause any health problems [60, 61]. In addition, perceived risks of tobacco use and intention to quit between the younger and the adult smokers may contribute for this tobacco use differences [62].

Occupationally, adults who were employed OR 8.86, 95% CI (3.11, 25.20); unemployed or retired OR 5.46 95% CI (1.69, 17.64) and homemaker OR 1.54, 95% CI (0.45, 5.30); are more likely to use tobacco products than a student, respectively. This finding is in agreement with a study conducted in India by Pramhakar et al. (2012) as being a student is less likely to use tobacco than unemployed and employed adults [63]. Similarly, widowed is less risk factor for tobacco use than another marital status including married, separated, single, and divorced (Table 6). However, Cho and his colleagues found that unmarried adults are more likely to use tobacco products than other marital status including married one [64].

Overall, the present study indicated that the prevalence of tobacco use among male adults is higher than female. Besides, the smoking prevalence of low wealth index adults is relatively higher. This is consistent with other studies conducted in Ethiopia [28, 65] that shows increasing trends of tobacco use in both sexes. However, tobacco use has health burdens and economic impacts. As the evidence showed, tobacco use causes various health problems such as cancer [66–70], cardiovascular disease [71], and respiratory diseases [72]. Globally, about 8 million people die each year from tobacco-related deaths with a high proportion in low and middle-income countries [7]. Non-communicable diseases (NCD) contribute 44% of death in Ethiopia [73] and tobacco-related deaths was estimated to 17 thousand in 2016 [15]. Now, the government of Ethiopia shows it commitment by ratifying the strongest tobacco bill in line with the WHOFCTC provisions to reduce public health impact of tobacco. Therefore, the regulatory, tobacco control actors, and all concerned bodies should implement all tobacco control laws and regulation without exceptions in order to curb tobacco epidemics and its economic burden.

## Conclusion

Although the prevalence of tobacco use is low compared to other African countries, the present study showed that the prevalence of tobacco use among adults is a concern that need special attention. Our study is the first to look at tobacco use and policy in Ethiopia alone. However, we have extensively reviewed several literatures to compare our findings with other studies. However, studies on the state of tobacco use at the national level are very limited. However, despite some differences in purpose and study methods; we compared our findings with DHS and STEP surveys to see changes. Accordingly, we obtained a consistent result. Our finding hence validates data consistent with the early studies of tobacco use in Ethiopia and raises concerns that smoking products including manufactured and shisha smoking may be more important, particularly among low economic groups, male adults and non-believer religion groups. The majority of adults used smoked products than smokeless tobacco. Factors such as gender, age, marital status, and occupation were significantly associated with the current tobacco use in Ethiopia. Regular and comprehensive tobacco control measures in line with WHO-MPOWER package are needed to reduce the spread of smoking among adults. In addition, the government of Ethiopia in collaboration with all stakeholders could design a specific tobacco control intervention that targets younger age groups, low economic groups, and all adults regardless of their educational status, residential area, and socioeconomic status.

## Data Availability

The datasets used and/or analyzed during the current study are available from the Global Tobacco Surveillance System database freely. The data also can be accessed by fulfilling the data sharing policy of the Ethiopian Public Health Institute.
